# Mitochondrial calcium buffering depends upon temperature and is associated with hypothermic neuroprotection against hypoxia-ischemia injury

**DOI:** 10.1371/journal.pone.0273677

**Published:** 2022-08-31

**Authors:** Sergey Sosunov, Arnav Bhutada, Zoya Niatsetskaya, Anatoly Starkov, Vadim Ten

**Affiliations:** Department of Pediatrics, Division of Neonatology, Robert Wood Johnson Medical School, Rutgers University, New Brunswick, NJ, United States of America; Medical University of South Carolina, UNITED STATES

## Abstract

Hypothermia (HT) is a standard of care in the management of hypoxic-ischemic brain injury (HI). However, therapeutic mechanisms of HT are not well understood. We found that at the temperature of 32°C, isolated brain mitochondria exhibited significantly greater resistance to an opening of calcium-induced permeability transition pore (mPTP), compared to 37°C. Mitochondrial calcium buffering capacity (mCBC) was linearly and inversely dependent upon temperature (25°C—37°C). Importantly, at 37°C cyclosporine A did not increase mCBC, but significantly increased mCBC at lower temperature. Because mPTP contributes to reperfusion injury, we hypothesized that HT protects brain by improvement of mitochondrial tolerance to mPTP activation. Immediately after HI-insult, isolated brain mitochondria demonstrated very poor mCBC. At 30 minutes of reperfusion, in mice recovered under normothermia (NT) or HT, mCBC significantly improved. However, at four hours of reperfusion, only NT mice exhibited secondary decline of mCBC. HT-mice maintained their recovered mCBC and this was associated with significant neuroprotection. Direct inverted dependence of mCBC upon temperature in vitro and significantly increased mitochondrial resistance to mPTP activation after therapeutic HT ex vivo suggest that hypothermia-driven inhibition of calcium-induced mitochondrial mPTP activation mechanistically contributes to the neuroprotection associated with hypothermia.

## Introduction

Perinatal asphyxia and hypoxia-ischemia brain injury (HI) remains one of the major causes of permanent neurological disability in children. The HI usually occurs due to a collapse of systemic circulation at or near birth or during neonatal period. Although, effective resuscitation and return of cerebral circulation ensures survival, often with a full or near-full neurological recovery, worldwide, perinatal asphyxia and HI brain injury are still account for 23% of all infant deaths [1]. According to World Health Organization, 25% of survivors after birth asphyxia are diagnosed with persistent neurological deficit [2, 3].

The mechanisms of neuronal injury and death following HI insult are not well understood to devise a specific mechanism-targeting neuroprotective strategy with proven clinical benefit. At the same time, hypothermia (HT) has been clinically proven to be beneficial and has become a standard of care for neonates at risk for HI brain injury. In spite of a wide clinical implementation, there is no clear understanding of therapeutic mechanisms of HT. This lack in mechanistic knowledge limits further clinical development of the most optimal (the initiation time, duration, extent, adjuvant strategy depending upon the stage of reperfusion, etc.) use of HT. On physiological level, HT decreases cerebral metabolic rate and improves coupling between metabolic demand and cerebral blood flow during reperfusion [4]. This is associated with attenuation of hyperemia phase in reperfusion [5]. On cellular and molecular levels, HT has been shown to limit a release of excitatory amino acids [6], glutamate-induced NO and reperfusion-associated reactive oxygen species (ROS) with inhibition of NMDA-receptors phosphorylation and oxidative stress [7–10]. There are numerous reports on anti-apoptotic [11–13] and anti-necrotic [12] effects of HT in neonatal HI brain injury models. In human neonates and animal models of HI brain injury, HT not only improves cerebral energy metabolism [14], but also attenuates an oxidative stress, neuroinflammation and cytotoxic cerebral edema [9, 15, 16]. Thus, published data suggest that post-ischemic HT addresses nearly all reported mechanisms driving the evolution of cerebral injury in reperfusion. However, at the same time, this multidirectional action of HT highlights our incomplete understanding of the primary therapeutic mechanisms by which HT attenuates cellular injury.

Following HI insult, secondary energy failure is viewed as a fundamental event in the evolution of brain damage in reperfusion injury [17]. One of the molecular mechanisms of secondary energy failure is delayed mitochondrial dysfunction due to activation of Ca^2+^ induced mitochondrial permeability transition pore (mPTP). Once activated, mPTP collapses a proton motive force, rendering mitochondria incapable of energy production [18]. In the model of cardiac arrest, compared to normothermia (NT), therapeutic HT was associated with limited mPTP activation in cerebral and heart mitochondria [19, 20]. Earlier, Golovach et al. has found a significant dependence of Ca^2+-^induced mitochondrial swelling upon temperature at three different Ca^2+^ concentrations which argues for direct effect of the temperature on permeabilization of mitochondrial matrix membrane [21]. The authors have identified a break-point in the Arrhenius plot at the temperature = 30–34°C, when at the temperature < 30°C activation energy of Ca^2+^ induced mPTP was 130 ± 20 kJ/mole, but dropped to 50 ± 9 kJ/mole at the temperature > 34 C. This work suggests that at the lower temperature, a significantly greater energy is required to induce mitochondrial membrane permeabilization with Ca^2+^ compared to that at the physiological temperature. We hypothesized that mitochondrial ability to retain/buffer Ca^2+^ and therefore prevent an activation of mPTP in their inner membranes is inversely dependent on temperature, being greater in hypothermia compared to normothermia (37°C). Therefore, HT-preserved/enhanced mitochondrial Ca^2+^ buffering capacity (mCBC) mechanistically drives neuroprotection via decreased mPTP activation.

## Materials and methods

### Animals and the model of HI brain injury

All animal protocols were approved by IACUC committee of Columbia University and Rutgers University in accordance with the AAALAC guidelines. The C57BL/6J neonatal mice of box sexes with their dams were purchased from Jackson Laboratories (Bar Harbor, ME). We used the Rice-Vannucci model of regional HI brain injury in rats [22], adapted to ten-day old (p10) neonatal mice [23]. Briefly, HI brain injury was induced by permanent ligation of the right common carotid artery under isoflurane anesthesia. After 90 minutes of recovery, mice were exposed to hypoxia (humidified 8% O_2_/ 92% N_2_, Tech Air Inc., NY) for 15 min in the hypoxic chamber placed in the temperature-controlled (37°C) neonatal isolette (Airshield Inc. NC). Ambient temperature during hypoxia was constantly monitored and kept at 37 ± 0.5°C. Following hypoxia, animals were randomly assigned to HT or NT group. Both groups were re-oxygenated with medical air. NT mice were kept at the ambient temperature of 32–33°C to maintain their rectal temperature = 37 ± 0.5°C. HT group was exposed for 30 minutes to the ambient temperature = 26°C (rectal temperature = 31.4 ± 0.34°C) followed by 30 minutes of gradual (2°C increase every 10 minutes) rewarming to the rectal temperature of 37 ± 0.4°C. In addition, we compared injury in mice exposed to hyperthermia (HyperT, rectal temperature = 39 ± 0.3°C, 30 minutes) with NT counterparts. The rectal temperature was measured at 30 and 60 min of re-oxygenation/reperfusion, using DIGI-sense temperature controller (Cole-Parmer, IL) equipped with RET-5 rectal probe for small animals (Physitemp Instruments LLC, NJ).

### Assessment of cerebral injury

At 24 hours or 7 days after HI injury, brains were collected and either sectioned and stained with tri-phenyl-tetrazolium chloride (TTC for at 24 hr assessment) or fixed in 4% paraformaldehyde in 0.1 M phosphate buffer at 4 °C overnight for assessment at 7 days. For TTC staining we used 1 mm coronal sections. For Nissl staining, using a vibratome (VT1000S, Leica Biosystems Inc., Buffalo Grove, IL) coronal sections (50 μm thick every 500 μm) from the entire brain were obtained. Digital images of the brains after both staining were traced (Adobe Photoshop 4.0.1) and analyzed with NIH image software by the investigator ‘blinded’ to the study groups. The extent of cerebral atrophy (Nissl stained brains) in the ipsilateral hemisphere were defined as a percentage of the residual tissue in relation to the contralateral hemisphere (100%). In TTC-stained samples, brain injury was defined as direct infarct volume expressed as a % of the ipsilateral hemisphere.

### Mitochondrial isolation and Ca^2+^ buffering capacity

In separate cohorts of HT and NT mice, cerebral non-synaptosomal mitochondria were isolated at 30 minutes and at four hours of reperfusion, as described [23]. The ipsilateral (right) hemispheres were homogenized in the isolation buffer (225 mM mannitol, 75 mM sucrose, 5 mM HEPES, 1 mM EGTA (pH 7.2), 0.1 mg/ml BSA) and centrifuged at 1,100 *g* for 2 min at 4°C. The supernatant was mixed with 80% percoll, overlayed on 10% percoll and centrifuged at 18,600 g for 10 min. Mitochondrial pellets were washed in sucrose buffer (250 mM sucrose, 5 mM HEPES, 100 μM EGTA (pH 7.2), 0.1 mg/ml BSA) and centrifuged at 10,000 *g* for 5 min at 4°C. The final pellet was re-suspended in sucrose buffer without BSA and used for functional assay.

### Mitochondrial Ca^2+^ buffering capacity

**Fig 1:**
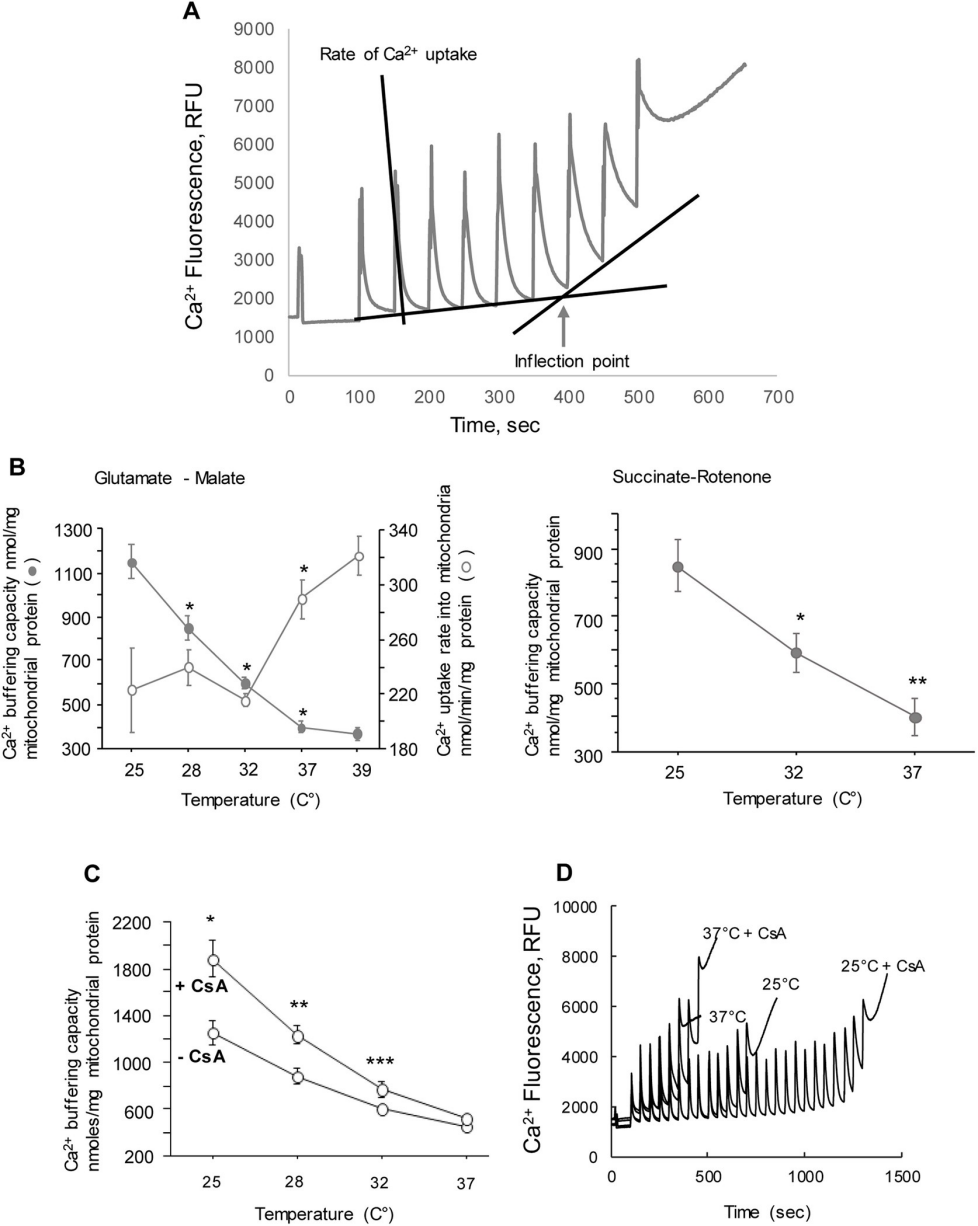
Ca^2+^ buffering capacity and the rate of mitochondrial Ca^2+^ uptake. **A**–Representative tracing of Ca^2+^ buffering capacity in isolated mitochondria with explanation of actual measurement of mCBC. B–Ca^2+^ buffering capacity (closed circle) measured with different substrates (indicated) and the rate of mitochondrial Ca^2+^ uptake (open circle) at various temperatures. * p < 0.01 and **p < 0.05 compared to the same metric obtained at the nearest lower temperature (for example mCBC at 28°C compared to that at 25°C, mCBC at 32°C compared to that at 28°C, etc.). For analysis of Ca^2+^ uptake rate (n = 5) and for the mCBC n = 10 at all temperature points, except at 39°C (n = 6) and at 25°C on Succinate-Rotenone (n = 11). **C and D**–Effect of CsA on mCBC at different temperatures with representative mCBC measurement tracings (**D**). * p = 0.002, ** p = 0.005 and *** p = 0.02 compared to the mCBC value without CsA. n = 6 in all experiments. RFU–relative fluorescence units.

To examine the relationship between mitochondrial Ca^2+^ buffering capacity and temperature, non-synaptosomal mitochondria were isolated from both hemispheres of naive p10 mice. Then, samples from three mice were combined to measure their mCBC at different temperatures: 25°C, 28°C, 32°C, 37°C and 39°C in the absence and presence of cyclosporine A (1μm, CsA). Mitochondrial Ca^2+^ buffering capacity was measured as described earlier [24, 25] with minimal modifications. In brief, mitochondria (0.05 mg/ml) were incubated in 10 mm Tris-MOPS buffer, pH 7.4, containing 120 mm KCl, 1 mm KH2PO4, 10 μm EGTA, 2 μm calcium green 5N, 5 mm malate and glutamate or 5 mM succinate and 1 μm rotenone. Mitochondria were given 5 nmoles of CaCl_2_ every 50 s. Mitochondrial Ca^2+^ uptake threshold (the point at which mitochondria start to lose the ability to take up all Ca^2+^ from the buffer) was determined by the inflection point in the line obtained by plotting the fluorescence levels after each Ca^2+^ addition (see Fig 1). Calculating the amount of Ca^2+^ per mg of mitochondrial protein required to reach the inflection point, we determined mitochondrial ability to buffer Ca^2+^ which prevents an opening of mPTP. Thus, total amount of Ca^2+^ sequestered by mitochondria at the inflection point was expressed as mitochondrial Ca^2+^ buffering capacity (mCBC).

### Statistical analysis

**Fig 2:**
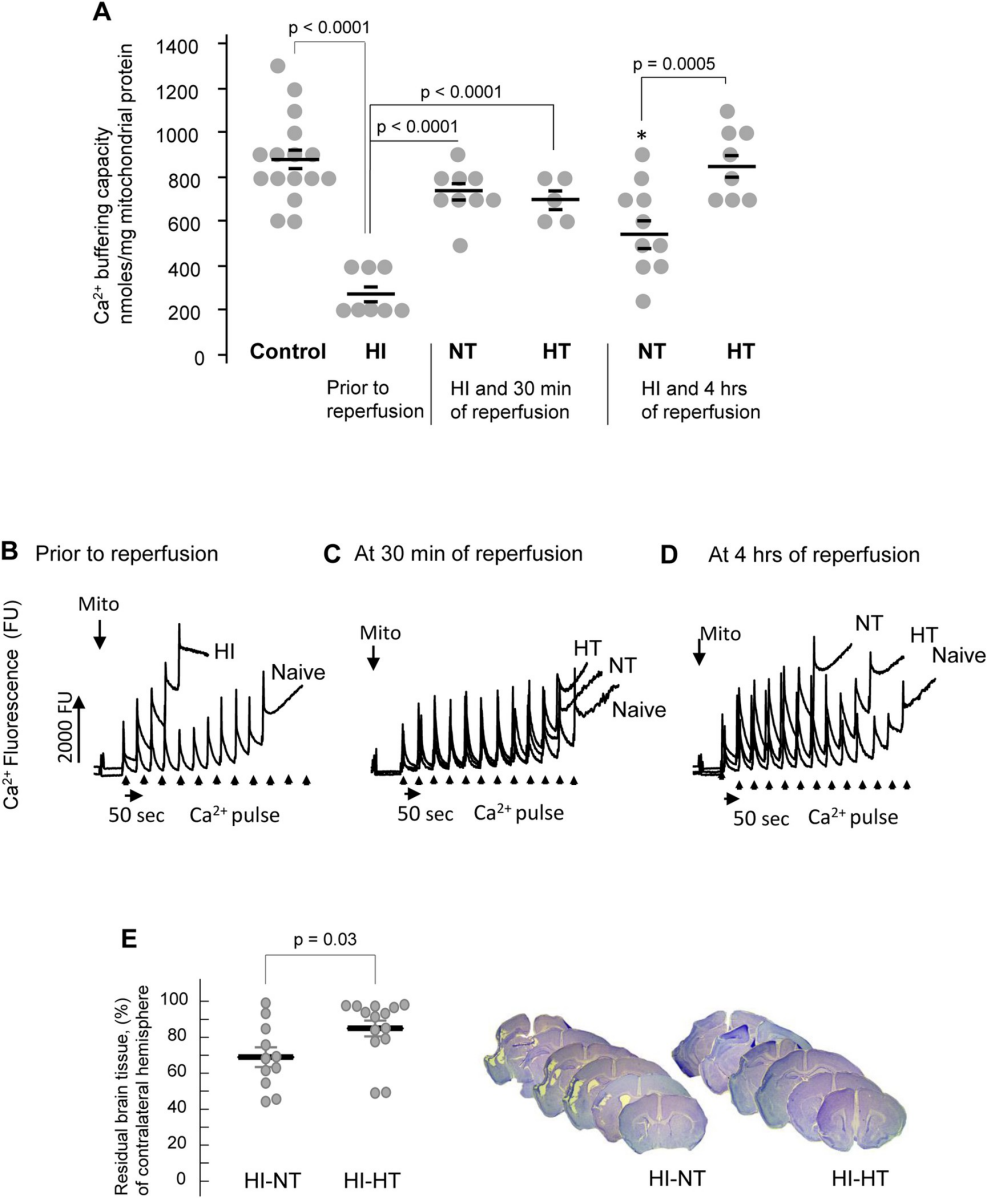
The protective effect of hypothermia in HI mice. **A**–Ca^2+^ buffering capacity in the brain mitochondria isolated at different time-points of reperfusion from HI mice exposed to NT or HT for the initial 30 minutes of reperfusion and compared to naïve controls. p-values are indicated. * p < 0.03 compared to 30 minutes NT group (Fisher’ post-hoc test). **B-D**—Representative tracings of mCBC obtained at different time of reperfusion (indicated) in mice exposed to HI insult and recovered either in NT or HT during the initial 30 minutes of reperfusion. **E**–Residual brain tissue volume assessed at seven days after HI insult in HI-mice exposed to NT or HT for the initial 30 minutes of reperfusion and representative Nissl-stained coronal sections.

All data were expressed as mean ± SEM. One-way ANOVA with Bonferroni’ or Fisher’ (where indicated) post-hoc analysis was used to determine a differences in the Fig 2A. Paired Student’s t-test was used to compare data shown in all other figures. The differences were considered significant if p value was ≤ 0.05.

## Results

### Mitochondrial calcium buffering capacity inversely and linearly depends on temperature

Mitochondria isolated from naïve neonatal p10 mouse brains and fueled with either NAD-linked (glutamate-malate) or FAD-linked (succinate) substrates demonstrated linear, reverse relationship between their Ca^2+^ buffering/retention capacity and temperature ranging from 25 to 37°C (Fig 1B). While mCBC was the greatest at 25°C and steeply and significantly decreased with an elevation of the temperature, no significant difference was detected between mCBCs measured at 37°C compared to 39°C (Fig 1B). Interestingly, analysis of intramitochondrial Ca^2+^ uptake rates revealed no difference in the rate of Ca^2+^ influx between the temperature ranges from 25°C to 32°C (Fig 1B). However, upon elevation the temperature from 32°C to 37°C the rate of Ca^2+^ influx significantly increased (Fig 1B).

### The effect of cyclosporine A depends on temperature

To determine if the mPTP activated in our experiments is sensitive to CsA, we tested mCBC with the presence or absence of CsA (1 μM). As expected, CsA significantly increased mCBC (Fig 1C and 1D), but only at 25°C. With elevation of the temperature, the effect of CsA gradually faded and at 37°C was minimal with no significant increase of mCBC (Fig 1C and 1D).

### Hypothermia prevented secondary drop in mCBC and attenuated HI brain injury

**Fig 3:**
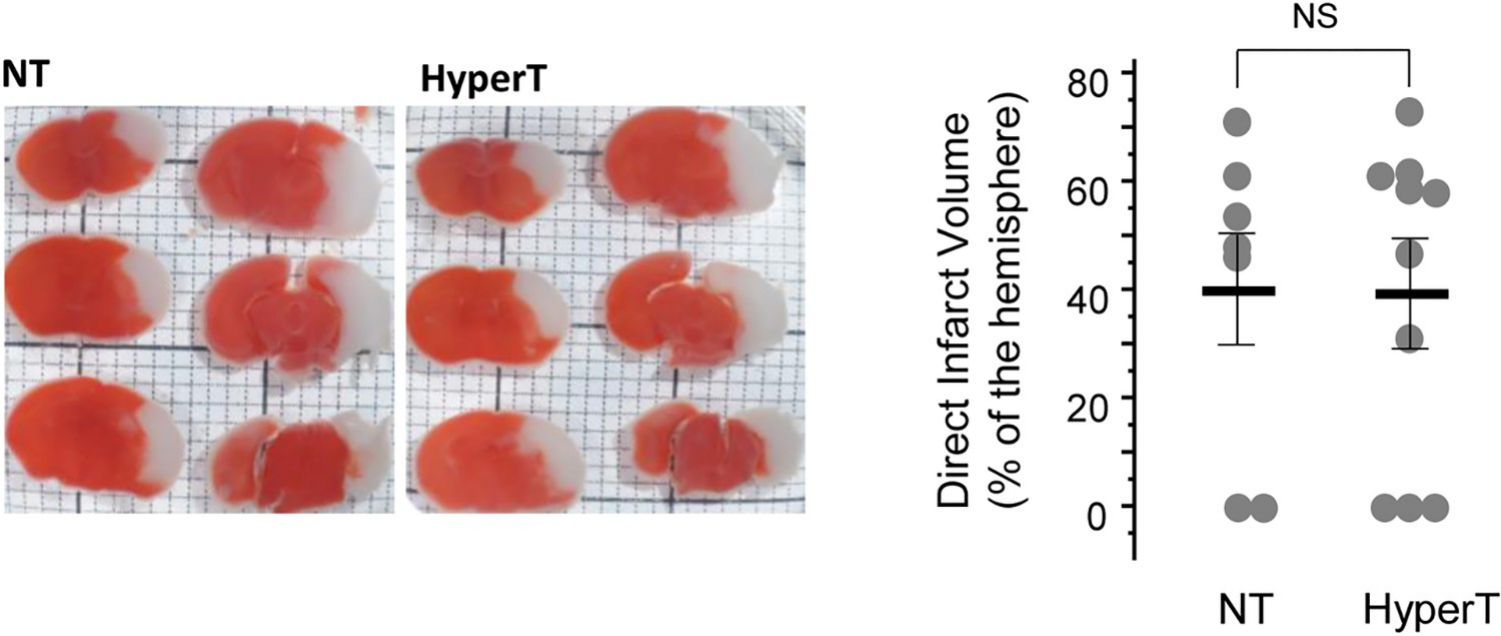
Hyperthermia in HI mice. Representative TTC-stained coronal brain sections obtained at 24 hours after HI from mice exposed to NT or Hyperthermia (HyperT) for the initial 30 minutes of reperfusion. NS–non significant.

Immediately after HI and prior to the reperfusion, mitochondrial Ca^2+^ retention capacity at 25°C was significantly decreased compared to controls (Fig 2A and 2B). At 30 minutes of normothermic or hypothermic reperfusion, in NT and HT mice, their mCBCs have significantly recovered and did not differ from that in controls (Fig 2A and 2C). At the later time-points of the reperfusion (4 hrs), mCBC again significantly decreased, but only in NT mice (Fig 2A and 2D). In contrast, post-ischemic mitochondria isolated from HT mice exhibited significantly greater mCBC compared to their normothermic counterparts (Fig 2A and 2D). Finally, the HT for the initial 30 minutes of reperfusion and 30 minutes of rewarming resulted in neuroprotection evidenced by a significantly greater preserved hemispheric tissue volume compared to that in NT mice (Fig 2E). Because there was no difference in mCBC measured at 37°C or 39°C (Fig 1B), we tested and determined that hyperthermia (rectal temperature = 39°C) for the initial 30 minutes of reperfusion and normalization to the rectal temperature of 37°C over the next 30 minutes did not affect the extent of the brain damage (Fig 3).

## Discussion

This is the first report demonstrating linear and inverse temperature-dependence of mPTP induction assessed by measuring Ca^2+^ buffering capacity. We have shown that *in vitro*, with lowering temperature from 37°C to 25°C mCBC increases dramatically and is significantly greater at 32°C compared to 37°C. Ca^2+^ induced activation of mPTP has been implicated in progression of ischemia-reperfusion brain injury in neonatal and mature animal models [25–28]. Therefore, our *in vitro* observation may highlight molecular mechanism of neuroprotection afforded by post-HI hypothermia. In patients cooled after cardiac arrest, the core temperature targeted during clinical hypothermia ranges between 32–34°C [29], and for neonates at risk for HI brain injury, recommended HT ranges between 33–34°C with reports on safe cooling down to 31°C [30, 31]. Earlier, a reversed dependence of mitochondrial swelling rate upon temperature has been reported [21]. Considering mitochondrial swelling as the evidence for mPTP activation, authors demonstrated that activation energy for opening of mPTP was 130 ± 20 kJ/mole at the temperatures below the break point of the Arrhenius plot (30–34°C) and 50 ± 9 kJ/mole at greater than 34°C. It is intriguing that the above referenced temperature-break point in the Arrhenius plot is nearly the same as the core temperature targeted during therapeutic HT. Our study using different methodology to probe organelles for their mPTP activation also showed a significant difference in mitochondrial tolerance to Ca^2+^-induced permeabilization at the temperature 32°C compared to that at 37°C. Although, mitochondrial swelling in the presence of Ca^2+^ could be considered as the evidence for mPTP activation, our work offers quantitative analysis of mitochondrial Ca^2+^ load (mCBC) required for mPTP opening at different temperature. Mitochondrial ability to uptake and store Ca^2+^ depends on the activity of respiratory chain to maintain a proton motive force and membrane potential. It has been shown that activation of CsA-sensitive mitochondrial ion/proton leak and mPTP opening causes depletion of mitochondrial NAD [32]. Theoretically, a gradual loss of NAD from the matrix during mitochondrial Ca^2+^ buffering capacity assay, may explain temperature-dependence of mCBC, as NAD depletion may occur faster at higher temperature. However, mitochondria fueled with succinate, a substrate linked to FAD which does not leaks-out with activation of mPTP, demonstrated the same linear dependence of mCBC upon temperature. This result suggests that a direct dependence of Ca^2+^ induced mPTP activation upon temperature and changes in mPTP activation energy upon temperature [21] are not driven by a loss of NAD and related dysfunction of respiratory chain.

Mitochondrial function to take up and buffer excessive cytosolic Ca^2+^ is critical for cellular survival and mitochondrial resistance to Ca^2+^-induced permeabilization of their inner membranes, the event known as mPTP [18, 28]. Once mPTP is activated, organelles collapse their membrane potential and loose a proton motive force for ATP production. This ensures cellular bioenergetics crisis. In the pathogenesis of acute cerebral ischemia-reperfusion injury, decreased mCBC was causally linked to cellular damage [26]. We asked if in the mouse model of neonatal HI brain injury, brief HT initiated at the onset of reperfusion affects mCBC in organelles isolated from the ischemic hemisphere, and if this is associated with attenuation of brain injury. HT initiated immediately after HI-insult significantly decreased the extent of brain injury assessed at seven days after the index event. Compared to NT-mice, this neuroprotection was associated with a significantly greater mCBC. Interestingly, in both NT and HT groups, immediately after HI-insult, mCBC was dramatically decreased compared to controls, but has recovered at 30 minutes of reperfusion to a similar extent. At four hours of reperfusion, while HT-mice fully preserved their recovered mCBC, NT mice exhibited a profound secondary drop in their mCBC, the event consistent with secondary energy failure. Earlier, we and others have reported that secondary energy/mitochondrial failure occurs at 4–5 hours of reperfusion in this model and in the model of fetal HI [25, 33]. In the model of middle cerebral artery occlusion, secondary mitochondrial failure occurred at 4–6 hours of reperfusion [34]. Thus, our data suggest that compared to the NT reperfusion, exposure to HT for the initial 30 minutes of reperfusion does not modify the initial recovery, but significantly greater preserves a recovered mCBC assessed during the time of reperfusion consistent with secondary energy failure. Given that mPTP activation is one of the widely reported mechanisms of secondary mitochondrial failure [33–35], a second decline of mCBC during NT-reperfusion in our study reflects mitochondrial susceptibility to Ca^2+^ induced mPTP activation. HT applied during the initial 30 minutes of reperfusion, makes organelles resistant to Ca^2+^ mPTP by preserving recovered mCBC. Because mice exposed to HT exhibited a significantly decreased extent of their brain injury and this was associated with near-normal mCBC at the stage of secondary energy failure, we propose that natural dependence of Ca^2+^ induced activation of mPTP upon temperature plays a mechanistic role in the neuroprotection afforded by HT. In the model of swine cardiac arrest, HT applied after a return of spontaneous circulation significantly greater preserved the integrity of isolated cortical mitochondria challenged by the exogenous Ca^2+^ [20]. Authors interpreted these data as the HT-induced attenuation of neuronal mPTP activation evidenced by decreased cytochrome-C and apoptosis-inducible factor release, activation of the caspase-3 and associated with improved neurological score. HT (31°C) protected cultured myocardiocytes against simulated ischemia-reperfusion and this effect was also associated with significantly decreased intra-mitochondrial Ca^2+^ load and preserved mitochondrial integrity [36]. HT-driven preservation of mCBC in HT mice in our study can be explained by the earlier referenced break point in the Arrhenius plot at 30–34°C. This break-point remained unchanged regardless of the difference in Ca^2+^ concentration [21], which suggests that temperature regulates mitochondrial inner membrane ability to withstand Ca^2+^ pore-forming effect. In addition, our data on the kinetic of Ca^2+^ influx demonstrated no difference in the rate of intramitochondrial Ca^2+^ intake at various temperature, except the temperature range from 32°C to 37°C. This suggests that temperature modifies not only mitochondrial matrix membranes properties, but also at the level consistent with the temperature targeted in therapeutic hypothermia modifies activity of Ca^2+^ uniporter and other Ca^2+^ pumping channels compared to that in the physiological temperature.

We realize that we do not show direct proof for mechanistic role of *in vitro* detected temperature-dependence of mCBC in HT-driven neuroprotection. However, our work demonstrates not only novel direct inverse dependence of mCBC upon temperature, but couples HT-driven neuroprotection with temperature*-*regulated mitochondrial ability to withstand Ca^2+^-induced activation of mPTP.

Importantly, no difference was detected in mCBC measured at the temperature of 37°C and 39°C. Thus, if the above proposed mechanistic link between temperature and mCBC indeed carries clinical significance, then hyperthermia exposure to a rectal temperature = 39°C should not change the extent of the brain damage compared to 37°C. Indeed, no difference in the infarct volume was detected in mice exposed for the initial 30 minutes of reperfusion to NT or to hyperthermia. This result strongly supports our hypothesis that HT affords neuroprotection by enhancement of mitochondrial tolerance to Ca^2+^ load.

The neuroprotection achieved by relatively brief (30 minutes) HT and 30 minutes of rewarming suggests that in our model temperature-dependent mitochondrial changes predisposing to mPTP formation occurs early, within an hour after the insult. Thus, to address this particular mechanism of cellular injury, the HT should be initiated immediately after HI. There is another important finding to discuss: Temperature dependent effect of CsA on mCBC. In contrast to that at 25°C– 32°C, at 37°C, CsA has failed to increase mCBC. This novel observation may have important translational utility explaining conflicting data on the efficacy of CsA therapy in neonatal HI brain injury model [37–39]. Furthermore, this finding highlights a direction for research on HT-potentiated therapeutic effects of anti-mPTP strategies.

Thus, our work offers novel *in vitro* data demonstrating: a) a tight linear and reversed dependence of mCBC upon a temperature ranged between 25 to 37°C, b) a loss of CsA effect on mCBC in the physiological temperature and c) significant increase in the rate of mitochondrial Ca^2+^ uptake driven by an elevation of temperature from 32°C (the core temperature targeted during therapeutic hypothermia) to 37°C. Considering a strong association between mCBC in the mice subjected to HT and neuroprotection, we believe that our *in vitro* discovery offers very important information for therapeutic mechanisms targeted by the HT applied at the initiation of reperfusion.
